# The Specific Judo Training Program Combined With the Whole Body Cryostimulation Induced an Increase of Serum Concentrations of Growth Factors and Changes in Amino Acid Profile in Professional Judokas

**DOI:** 10.3389/fphys.2021.627657

**Published:** 2021-02-09

**Authors:** Joanna Jaworska, Radoslaw Laskowski, Ewa Ziemann, Klaudia Zuczek, Giovanni Lombardi, Jedrzej Antosiewicz, Piotr Zurek

**Affiliations:** ^1^Department of Physical Education and Lifelong Sports, Poznań University of Physical Education, Poznań, Poland; ^2^Department of Physiology and Biochemistry, Gdańsk University of Physical Education and Sport, Gdańsk, Poland; ^3^Department of Athletics, Strength and Conditioning, Poznań University of Physical Education, Poznań, Poland; ^4^Department of Medical Biology and Genetics, Faculty of Biology, University of Gdańsk, Gdańsk, Poland; ^5^IRCCS Galeazzi Orthopaedic Institute, Lab Experimental Biochemistry & Molecular Biology, Milan, Italy; ^6^Department of Bioenergetics and Physiology of Exercise, Medical University of Gdańsk, Gdańsk, Poland; ^7^Department of Physical Culture Gorzow Wielkopolski, Poznań University of Physical Education, Poznań, Poland

**Keywords:** physical therapy, martial arts, irisin, interleukin 15, recovery

## Abstract

This study aimed to evaluate the effect of a specific training program, supported by 10 sessions of whole body cryostimulation, on growth factors concentrations, amino acids profile and motor abilities in professional judokas. Ultimately, twelve athletes took part in the study. They were randomly assigned to the cryostimulation group (CRY, *n* = 6) or the control group (CON, *n* = 6). During 2 weeks of the judo training program, the CRY group performed 10 cryo-sessions (3-min, at a temperature of −110°C) and the CON group rested passively. Anthropometric measurements, a strength test, the Special Judo Efficiency Test (SJET) were assessed 2 days before and after the judo training program. Blood samples were collected at rest, 1 h after the first and the second SJET and 1 h after the first and the last cryo-session to establish growth factors and amino acid concentrations. Lactate level was measured before, immediately after and 1 h after the first and the second SJET. The applied intervention resulted in a significant increase of resting concentrations of brain-derived neurotrophic factor (from 10.23 ± 1.61 to 15.13 ± 2.93 ng⋅ml^–1^; *p* = 0.01) and insulin-like growth factor 1 (IGF-1; from 174.29 ± 49.34 to 300.50 ± 43.80 pg⋅ml^–1^; *p* = 0.00) in the CRY group. A different response was registered 1 h directly post SJET in the CRY group (a significant increase of IGF-1, interleukin 15 and irisin: *p* = 0.01; *p* = 0.00; *p* = 0.03). Additionally, the significant drop of proline and leucine concentrations in the CRY group was obtained. Athletes’ performance remained unchanged in both groups. However, subjects perceived positive changes induced by the intervention – not directly after cryostimulation but in response to the specific training workload. The increase of growth factors concentrations and the improvement of amino acid profile (proline and leucine) contributed to maintaining a high level of muscle function.

## Introduction

Competitive judo is a demanding Olympic discipline, which requires a high level of technical and tactical skills (Franchini et al., 2008) as well as physical fitness (Franchini et al., 2011). Judokas follow various complex training programs, geared toward technical and tactical practice, traditional strength and endurance training as well as fight simulation such as *Randori* (Franchini and Takito, 2014), which evoke muscle damage even among experienced judo fighters (Laskowski et al., 2011). In the last few years, the frequency of judo competitions has increased significantly (Sikorski, 2011). Athletes thus face the challenge of maintaining a steady, high level of physical performance and decision-making skills for an extended period (Garatachea et al., 2012).

The specificity of the judo training process can pose a challenge for coaches and physiotherapists as they need to determine an optimal training program for their athletes, involving appropriate recovery methods. Meanwhile, our knowledge of the recovery methods adequate for judokas is lacking, particularly in the aspects of fostering adaptation to a judo training program or tailoring recovery to athletes’ special needs such as eliminating chronic injuries, aiding progressive weight loss or maintaining muscle mass size. At the same time, deficient recovery practices within a professional training program, may lead to functional overreaching and, in consequence, trigger an overtraining syndrome (Meeusen et al., 2013).

Cold therapy is one of the most popular recovery methods used by athletes (Murray and Cardinale, 2015; Lombardi et al., 2017b). However, literature data have not unequivocally verified its impact on regeneration, physical performance and muscle adaptation. On the one hand, cold treatment was demonstrated to enhance the muscle recovery process and attenuate muscle damage including delayed-onset muscle soreness (DOMS) (Siqueira et al., 2018). On the other hand, its application does not always have a positive effect on muscle recovery or a visible impact on the recovery index (Malta et al., 2018). This differentiated effect of cold therapy depends on the applied procedure, the type of training and tasks executed simultaneously. Attenuated anabolic signaling and muscle hypertrophy in response to cold water immersion (CWI) was mainly recorded after a one-repetition exercise test and less so after regular training or endurance exercise (Malta et al., 2021). Interchangeably with CWI, whole body cryostimulation or partial exposure to extremely low temperature is also applied. Although the increasing accessibility of cryo-chambers is making the implementation of whole body cryostimulation in training more feasible and convenient, reports on the application of this treatment as part of a professional training program are limited (Rose et al., 2017). Therapies based on exposure to extremely low temperatures (−110 to −130°C) are known to be well-tolerated by professional athletes (Bouzigon et al., 2018), to limit exercise-associated inflammation and to enhance athletes’ performance during demanding competition and training periods (Ziemann et al., 2012; Schaal et al., 2015; Bouzigon et al., 2020). Positive impact on levels of growth factors such as brain-derived neurotrophic factor (BDNF) or insulin-like growth factor (IGF-1), and blood concentrations of amino acids (Jaworska et al., 2018), linked with this temperature range, have been recorded mainly in academic, non-professional athletes. Therefore, the question of the effectiveness and validity of applying this kind of recovery strategy in professional training remains open.

Therefore, this study aimed to investigate the effect of 10 sessions of whole body cryostimulation on athletes’ performance, levels of growth factors and amino acid concentrations in professional judokas during a 2-week high-intensity specific judo training program.

## Materials and Methods

### Study Design

**FIGURE 1:**
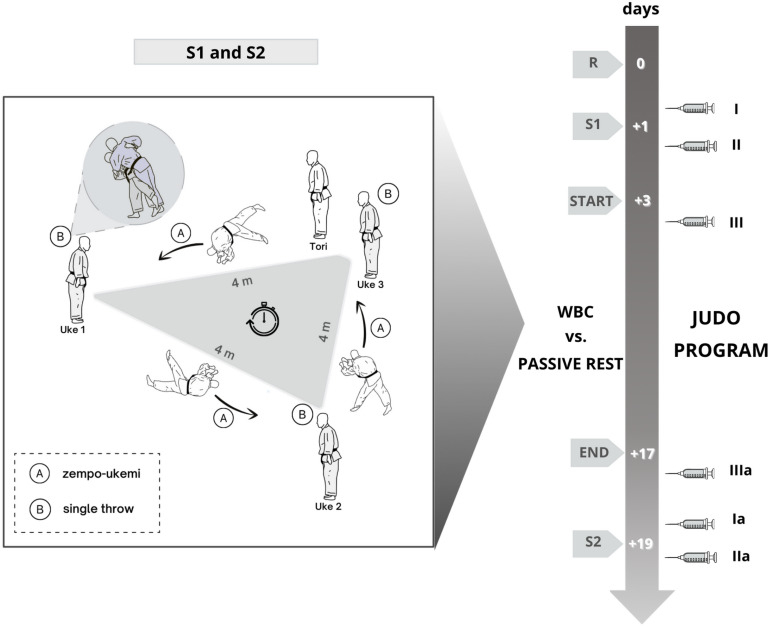
The study schedule and the configuration of the Special Judo Efficiency Test (SJET). R – recruitment and division judokas into two group, WBC – whole body cryostimulation, S1: blood samples collection (syringe): at rest (I, Ia), anthropometric measurements, strength test, the Special Judo Efficiency Test (SJET) before (S1) and after intervention (S2). Blood samples collection 1 h after the first (II) and the second (IIa) SJET; 1 h after the first (III) and last cryo-session (IIIa). The SJET illustration: **(A)** – “zempo-ukemi” judo forward roll break-fall performed by Tori (thrower) toward other Uke (throw receiver), **(B)** – single throw performed by Tori on Uke, 4 m – distance between uke, timer – time of the test for men 5 min and for women 4 min.

The study design and timeline are presented in Figure 1. Professional judokas participated in a 2-week judo training program including various kinds of high intensity practices. In addition, the experimental group underwent 10 sessions of whole body cryostimulation, while the control group did not use any dedicated methods of recovery. 2 days prior to beginning the intervention and 2 days after its completion, blood samples were collected, anthropometric measurements were taken, muscle performance was assessed and the Special Judo Efficiency Test (SJET) was performed (Laskowski, 2007).

### Subjects

Thirteen highly trained judokas (all members of the Polish National Judo Team, age: 21 ± 3 years, height: 176 ± 8 cm, body weight 84 ± 22 kg, skeletal muscle mass 34 ± 7 kg, body fat 22 ± 14 kg, VO_2_max 47 ± 11 ml⋅kg^–1^⋅min^–1^) took part in the study. The average training experience of each judoka amounted to 10 ± 2 years. Athletes were randomly split into two groups: the experimental group (CRY; *n* = 7) and the control group (CON; *n* = 6). Subjects stayed in a university dorm and followed a predetermined, balanced diet, taking their meals in the same place and at the same time of day (daily average intake of 3,600 ± 600 calories included protein at 1.5 g⋅kg^–1^ body mass). This approach was based on the previously published protocol (Laskowski and Antosiewicz, 2003). None of the participants had any previous experience with whole body cryostimulation, and did not take any supplementation or prescribed drugs during the intervention. Subjects were informed about the risks associated with the study and provided a written consent to their participation. The Bioethical Committee of the Regional Medical Society in Gdańsk approved the investigation before its onset (KB-28/17). The study was conducted under the Declaration of Helsinki. Athletes knew well the venue where all tests, the training protocol and recovery sessions took place.

### Anthropometric Measurements

Body mass and body composition were assessed using a multi–frequency impedance body composition analyser (In Body 720, Biospace, Korea). Impedance of segments of the body parts (trunk, arms, and legs) was measured at diverse six frequencies (1, 5, 50, 250,500, and 1000 kHz), using an eight-polar tactile electrode (Volgyi et al., 2008). Measurements were taken according to the manufacturer’s protocol (McLester et al., 2020), following an overnight fasting (12 h after the last meal and drink). Athletes were also instructed to drink 400 ml of water, additional to their daily intake, and avoid any physical exercise 1 day before to the measurements.

### The Assessment of Muscle Performance

Muscle function of the lower and upper limbs was estimated using a Biodex System four dynamometer (Biodex Medical System, Inc. Medical System, NY, United States). After 10 min of standardized warm-up, athletes were positioned in the equipment, according to the manufacturer’s manual. Each test was performed in a sitting position and belts were used for body stabilization. Athletes received standardized verbal instructions before each test, and verbal encouragements throughout it. Subjects were asked to contract “as hard as possible” (to their maximum) to obtain their maximal peak torque. First, we measured muscle strength in isometric conditions, and recorded the peak contraction during flexion and extension in the knee and shoulder joints (of dominant limbs) in the conditions of 5-s isometric contractions. Then, we immediately measured the peak torque for the flexion and extension of the knee and shoulder joints (of dominant limbs) in the isokinetic conditions (velocity 90°⋅s^–1^) in a three-time repeated movement. This assessment was repeated twice before and after the whole training program intervention.

### The Assessment of Specific Physical Performance

The SJET was used to establish participants’ specific judo performance abilities (Laskowski, 2007). The configuration of the SJET is shown in Figure 1. During the test, judokas performed an effort similar to a competitive match, characterized by the high-intensity, the approximate number of actions and changing body positions (standing “nage-komi” – repetitive throwing practice with a partner executing the technique and groundwork position; “zempo-ukemi” – judo forward roll break-fall). The SJET was based on performing the highest number of throws in 5 min for men and 4 min for women. Before the test, athletes performed a standardized 15-min warm-up. During the SJET, subjects received verbal encouragements. SJET involved four judokas – one performing the throws and three athletes partnering (partners had a similar height and body mass as the athlete performing the test). Before every single throw, “zempo-ukemi” toward other judokas was done (Figure 1). Judokas’ heart rate during the SJET was measured using a Polar Team^2^; Pro device (Polar, Kempele, Finland).

### Training Program

The training protocol was conducted at the beginning of the training preparation phase. Every judoka attended two training sessions a day with various loads applied, including special judo technical training (standing position – *tachi waza* and ground position – *ne waza*) as well as endurance and resistance training components. Each training session begun with 15–20 min of warm-up and ended with 15 min of stretching exercises. The endurance training units consisted of a continued 50-min run with the intensity of 60% of maximum heart rate (MHR). Sprint training units performed at the track included sprints at 100, 200, 400, and 800 m (90% MHR). The whole-body resistance training focused on strength improvement and included four main exercises: clean and jerk, squat, bench press and barbell row. Each training consisted of five sets of five repetitions maximum (RM) of each exercise. During the current intervention athletes were under the strict control of the team’s coach (seven DAN judo, master class trainer) and a physiologist. The detailed schedule of the training program is shown in Table 1.

**TABLE 1:** The judo specific training protocol.

Day	Training at 10 am	Intensity	Recovery 12–1 pm	Training at 4 pm	Intensity
Monday	**A**	60–70% MHR	CRY/PR	**B**	60–70% MHR
Tuesday	**C**	90% MHR	CRY/PR	**D**	5 × 5 RM
Wednesday	**E**	60% MHR	CRY/PR	**F**	80–90% MHR
Thursday	**A**	60–70% MHR	CRY/PR	**B**	60–70% MHR
Friday	**C**	90% MHR	CRY/PR	**D**	5 × 5 RM
Saturday	**E**	60% MHR		Rest	
**Sunday**	**Rest**				
Mondaycy i	**A**	60–70% MHR	CRY/PR	**B**	60–70% MHR
Tuesday	**C**	90% MHR	CRY/PR	**D**	5 × 5 RM
Wednesday	**D**	5 × 5 RM	CRY/PR	**F**	80–90% MHR
Thursday	**A**	60–70% MHR	CRY/PR	**B**	60–70% MHR
Friday	**C**	90% MHR	CRY/PR	**D**	5 × 5 RM
Saturday	**E**	60% MHR		Rest	

*CRY, single session of cryostimulation; PR, passive rest; MHR, maximal hart rate; RM, repetitions maximum. Training **A:** special judo training *ne-waza* base on repetitive technical exercise; 90 min. Training **B:** special judo training *tachi-waza* based on repetitive throwing exercise; 90 min. Training **C:** high-intensity sprint training at the running track, 100, 200, 400, and 800 m; 60 min. Training **D:** whole-body resistance training at the gym; four exercise, five sets of 5 RM, 2 min break between sets; 60 min. Training **E:** endurance training based on continuous moderate-intensity run; 50 min. Training **F:** fight practice – *Randori*; four sparring in *ne-waza* and 8 sparring in *tachi-waza*, every lasted 5 min, 2 min break between fight; 120 min.*

### Whole Body Cryostimulation

All athletes in the CRY group participated in 10 sessions of whole body cryostimulation in a cryogenic chamber (Zimmer, Medizine-systeme, German) at the Pomeranian Rheumatologic Centre in Sopot, Poland. Sessions took place between 12 and 1 pm from Monday to Friday under medical supervision. Every session lasted 3 min. Before entering the main cryo-chamber, participants followed the adaptation procedure in the vestibule at a temperature of −60°C (approx. 30 s). The temperature in the main cryogenic chamber was −110°C. During the treatment, participants wore only shorts, socks, gloves and hats to cover their auricles. The sessions were conducted following the standardized protocol of whole body cryostimulation (Lombardi et al., 2017b).

### Blood Collection and Analysis

In order to assess growth factors concentrations, blood samples were collected 2 days before and after the specific training program, at baseline and 1 h after the first and second SJET test (Figure 1). Additionally, blood samples were taken 1 h after the first and last cryo-session. Samples were taken from the antecubital vein into vacutainer tubes with K2EDTA (Becton Dickinson and Co., Franklin Lakes, NJ, United States) by professional medical staff. Immediately following blood collection, samples were centrifuged at 2,000*g* for 10 min at 4°C and the serum was stored at −80°C until later analysis, according to the currently available pre-analytical warnings (Lombardi et al., 2017a).

For blood lactate concentration (LA) analysis, samples were collected from capillary blood taken from the right index fingertip before the warm-up, immediately after and 1 h after the first and the second SJET. Directly after the collection, samples were deproteinized adding ice-cold 0.4 M perchloric acid. After being thoroughly mixed, samples were centrifuged at 12,000*g* for 10 min. Blood LA was determined using a standard Randox (Crumlin, United Kingdom) kit based on the LA oxidase method (LC2389). Assays were performed on the Cecil CE9200 spectrophotometer (Cambridge, United Kingdom).

Serum concentrations of BDNF, IGF-1 and interleukin-15 (IL-15) were assessed using sandwich ELISA kits according to manufacturer’s instructions (R&D System, United States; catalog no. DBD00, DG100, and D1500, respectively). Irisin concentration too was determined with an ELISA kit (Phoenix Pharmaceutical Inc. United States; catalog no. EK 067-16).

Quantification of serum amino acids was done through the ion-pair reversed phase high performance liquid chromatography combined with the tandem mass spectrometry IP-RP HPLC-MS/MS triple state quadrupole (TSQ Vantage Thermo Scientific) and was performed following the same procedure as in the previous study (Gmiat et al., 2018).

### Statistical Analysis

Statistical analysis was performed using Statistica 13.1 software. All values are expressed as a mean ± standard deviation (SD). Shapiro–Wilk test was used to assess the homogeneity of dispersion from the normal distribution. Brown–Forsythe test was used to evaluate the homogeneity of variance. For homogenous results, the analysis of variance (ANOVA) for repeated measures and the *post-hoc* Tukey’s test for unequal sample sizes were performed to identify significantly different results. For heterogenous results, ANOVA Friedman’s test and Dunn-Bonferroni *post-hoc* test were used. The effect size (partial eta squared, η${}_{p}^{2}$) was also calculated, with η${}_{p}^{2}$ ≥ 0.01 indicating small effect; ≥0.059 indicating medium effect; and ≥0.138 indicating large effect (Cohen, 1988). The significance level was set at *p* < 0.05. Additionally, due to the small size of the study group, all measurements were compiled in a spreadsheet for the analysis of parallel-group trials and the effects were interpreted using the magnitude-based inferences decision. All data was log-converted to reduce bias arising from the error non-uniformity. Probabilistic conclusions about the true (large-sample) value of effects were provided in the spreadsheet as clinical magnitude-based inferences (Hopkins et al., 2009). We expressed uncertainty in each effect as 90% confidence limits and as probabilities that the true effect was beneficial (e.g., a substantial increase in irisin level) and harmful (e.g., a substantial decrease in irisin level). Clinically clear beneficial effects were those for which the benefit was at least possible (>25% chance) and the risk of harm was acceptably low (<0.5%). Effects where the chance of benefit outweighed the risk of harm (odds ratio of benefit to harm >66) were also deemed clear. Other effects were either clearly non-beneficial (chance of benefit <25%) or unclear (chance of benefit >25% and risk of harm >0.5%). Clear effects were reported as the magnitude of the observed value, with the qualitative probability that the true effect was beneficial, trivial or harmful for the change (e.g., in irisin level). The scale for interpreting the probabilities was as follows: ^∗^ – possible, ^∗∗^ – likely, ^∗∗∗^ – very likely, ^****^ – most likely (Hopkins et al., 2009).

## Results

### Subjects

Twelve athletes completed the intervention. One judoka from the CRY group was excluded from the study due to his injury (CRY, *n* = 6; CON, *n* = 6). After the intervention, no changes in body composition or body mass were recorded (data not shown).

### Changes in Physical Performance

Muscle performance test data indicated that judo training did not affect athletes’ muscle function, regardless of the applied recovery strategy. Among all analyzed parameters characterizing muscle function no significant changes were noted. Parameters remained unchanged, regardless of the rest procedure (Table 2). Results of the SJET also did not differ significantly. The average number of throws in the first SJET reached 57 ± 14 (CRY 53 ± 12; CON 61 ± 15) with the average HR during the test 183 ± 7 bpm (CRY 183 ± 5; CON 185 ± 6). After the 2-week training program, the average number of throws increased slightly in the second SJET and totaled at 60 ± 14 (CRY 56 ± 11; CON 63 ± 15) with the average HR 180 ± 3 bpm (CRY 181 ± 1; CON 179 ± 4).

**TABLE 2:** The muscle functions results before and after 2-week of specific judo training program combined with 10 sessions of cryostimulation or passive rest in both groups.

		PRE		POST		SMD	MBI	ANOVA *p* (η${}_{p}^{2}$)
		$\bar{X}$ ± SD	95% CI	$\bar{X}$ ± SD	95% CI	d – Cohena	Practically worthwhile effect	Group × Time Interaction
**PT max isometric [Nm] extension**								
Dominant leg	CON	348.47 ± 74.42	270.37– 426.56	393.62 ± 125.85	261.55–525.69	0.44	Unclear	0.12 (0.23)
	CRY	306.63 ± 89.71	212.48 – 400.77	307.16 ± 99.38	202.87–411.46	0.01		
Dominant shoulder	CON	135.22 ± 43.69	89.37–181.07	137.38 ± 41.06	79.51–193.32	0.05	Unclear	0.99 (0.00)
	CRY	131.63 ± 47.32	81.97– 181.29	133.93 ± 49.98	81.48–186.38	0.05		
**PT max isometric [Nm] flexion**								
Dominant leg	CON	153.43 ± 29.83	122.13–184.74	157.59 ± 37.15	118.60–196.58	0.12	Unclear	0.43 (0.06)
	CRY	155.20 ± 32.95	120.61– 189.78	149.46 ± 24.68	123.56– 175.36	0.20		
Dominant shoulder	CON	105.01 ± 38.58	64.52–145.49	98.50 ± 23.49	73.85–123.15	0.20	Unclear	0.98 (0.06)
	CRY	94.27 ± 28.72	64.14– 124.41	95.54 ± 35.51	58.27– 132.81	0.04		
**PT max isokinetic 90°• s^–1^ [Nm] extension**								
Dominant leg	CON	245.36 ± 43.94	199.24–291.47	229.16 ± 35.11	192.31–266.00	0.41	Unclear	0.59 (0.03)
	CRY	235.12 ± 64.49	167.44– 302.80	224.64 ± 60.09	161.58– 287.70	0.17		
Dominant shoulder	CON	107.83 ± 31.55	74.72–140.94	111.63 ± 23.67	86.79–136.47	0.14	Unclear	0.33 (0.01)
	CRY	98.32 ± 33.59	63.07–133.57	98.75 ± 29.78	67.50– 130.00	0.01		
**PT max isokinetic 90°• s^–1^ [Nm] flexion**								
Dominant leg	CON	137.91 ± 21.92	114.91–160.91	129.17 ± 17.50	110.80–147.53	0.44	Unclear	0.44 (0.06)
	CRY	136.35 ± 35.58	99.02– 173.69	134.33 ± 38.00	94.45– 174.22	0.05		
Dominant shoulder	CON	96.40 ± 23.48	71.76–121.04	96.60 ± 13.46	82.48–110.73	0.01	Unclear	0.61 (0.02)
	CRY	84.92 ± 28.01	55.53– 114.31	82.07 ± 25.70	55.10– 109.04	0.11		
**AP max isokinetic 90°• s^–1^ [W] extension**								
Dominant leg	CON	147.07 ± 57.69	86.52– 207.61	149.87 ± 10.61	138.73–161.00	0.07	Unclear	0.54 (0.04)
	CRY	167.95 ± 48.49	117.06–218.84	157.43 ± 40.27	115.17– 199.69	0.24		
Dominant shoulder	CON	85.25 ± 27.59	56.30– 114.20	80.27 ± 23.65	55.44–105.09	0.19	Unclear	0.12 (0.02)
	CRY	69.32 ± 29.15	38.72–99.91	69.70 ± 29.31	38.94–100.46	0.01		
**AP max isokinetic 90°• s^–1^ [W] flexion**								
Dominant leg	CON	101.42 ± 32.64	67.16– 135.67	106.25 ± 16.48	88.96– 123.54	0.19	Unclear	0.70 (0.02)
	CRY	108.98 ± 30.13	77.37– 140.60	108.43 ± 32.39	74.44– 142.42	0.02		
Dominant shoulder	CON	87.77 ± 36.88	49.07– 126.47	71.55 ± 17.75	52.92–90.18	0.56	Unclear	0.09 (0.07)
	CRY	64.25 ± 24.40	38.64–89.86	62.60 ± 23.88	37.54–87.66	0.07		

*PRE, before 2-week of special judo training program; POST, after 2-week of special judo training program; PT max, peak torque maximal, AP max, average power maximal; CRY, cryostimulation group; CON, control group; 95%CI – confidence interval; η ${}_{p}^{2}$, effect size, partial eta-squared; SMD, standardized mean difference; MBI, Magnitude-based inference.*

### Changes in Blood Lactate Concentration

The fatigue level was expressed by blood LA. Recorded values indicated that the intensity of SJET was high and above the anaerobic threshold for both groups. The average blood LA concentration immediately reached 7.06 ± 1.23 mmol⋅l^–1^ in the CRY group and 10.48 ± 1.86 mmol⋅l^–1^ in the CON group. ΔLA (the difference between the LA concentration recorded immediately after the SJET and 1 h rest) after the first SJET was 4.47 ± 1.02 mmol⋅l^–1^ in the CRY group and 4.37 ± 0.86 mmol⋅l^–1^ in the CON group. After the 2-week intervention, ΔLA were 3.74 ± 1.32 mmol⋅l^–1^ in the CRY group and 5.72 ± 1.49 mmol⋅l^–1^ in the CON group.

### Changes in Growth Factors Levels

**FIGURE 2:**
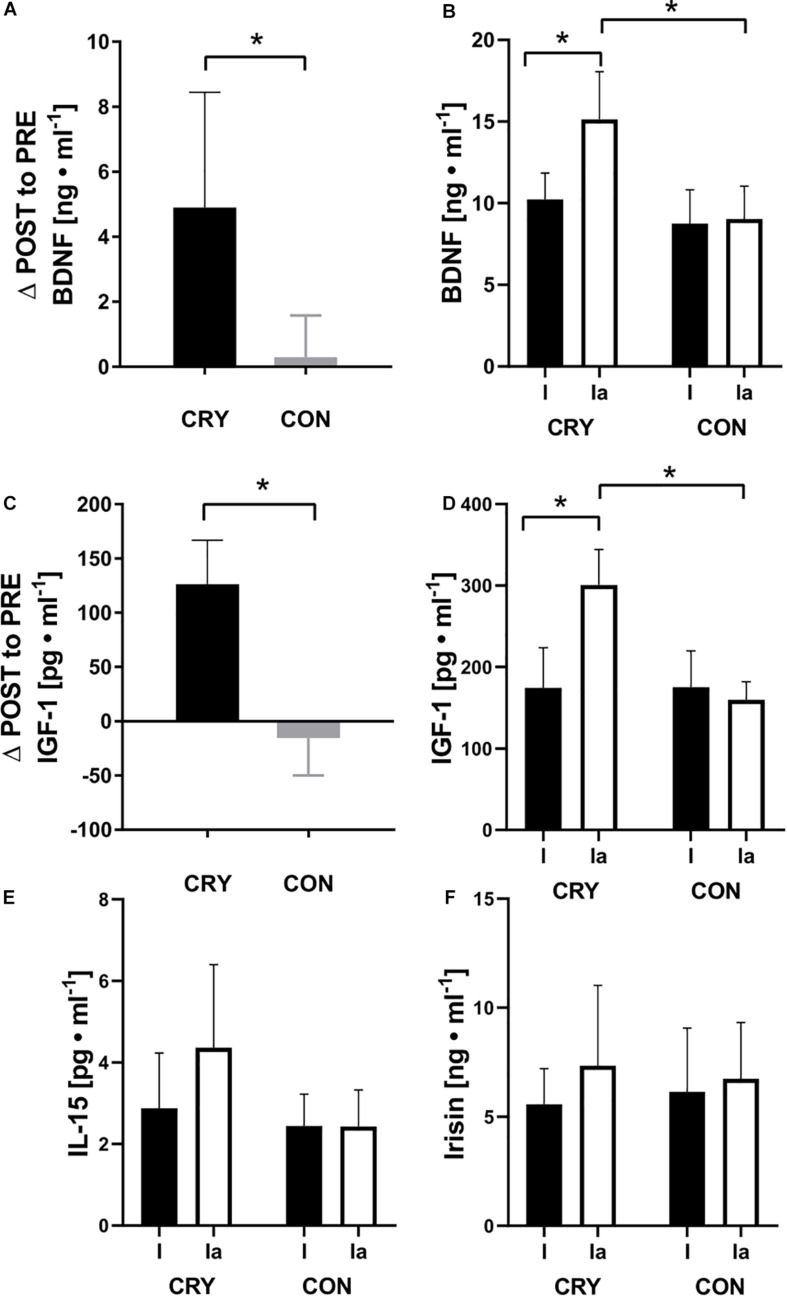
The immunological response induced by training and whole body cryostimulation. **(A)** delta changes (Δ POST to PRE) of brain-derived neurotrophic factor BDNF (effect size *p* η${}_{p}^{2}$ 0.47 and **(C)** insulin-like growth factor 1 – IGF-1 (effect size *p* η${}_{p}^{2}$ 0.81); comparison of serum growth factors concentration at rest: **(B)** BDNF, **(D)** IGF-1, **(E)** IL-15, **(F)** irisin; *significant changes (*p* < 0.05); CRY, cryostimulation group; CON, control group; POST, post intervention values, PRE, pre-intervention values; I, baseline, Ia, after 2-week the training program.

Changes in resting serum growth factors concentrations before and after the intervention are presented in Figure 2. The applied whole body cryostimulation, resulted in a significant increase of resting BDNF concentration from 10.23 ± 1.61 to 15.13 ± 2.93 ng⋅ml^–1^ (*p* = 0.01; Figure 2B). This change was accompanied by a significant elevation of IGF-1 concentration in the CRY group (pre: 174.29 ± 49.34; post: 300.50 ± 43.80 pg⋅ml^–1^; p = 0.00; Figure 2D). The effect size *p* $\left(\eta_{\rho }^{2}\right)$ of delta values recorded pre vs post of resting concentration of BDNF was 0.47 and, respectively, 0.81 of IGF-1. In the CON group, no changes in BDNF (pre: 8.74 ± 2.08 ng⋅ml^–1^ vs post: 9.04 ± 2.01 ng⋅ml^–1^) and IGF-1 (pre: 175.19 ± 44.67 pg⋅ml^–1^, post: 159.70 ± 22.33 pg⋅ml^–1^) were recorded. At the same time, only a slightly elevated tendency of IL-15 concentration was noted in both groups (Figure 2E). Independently of the applied recovery method, the training program did not alter resting irisin concentration (Figure 2F). Interestingly, a correlation between the resting level of irisin and ΔLA after the second SJET was noted (*r* = 0.90, *p* < 0.05).

**FIGURE 3:**
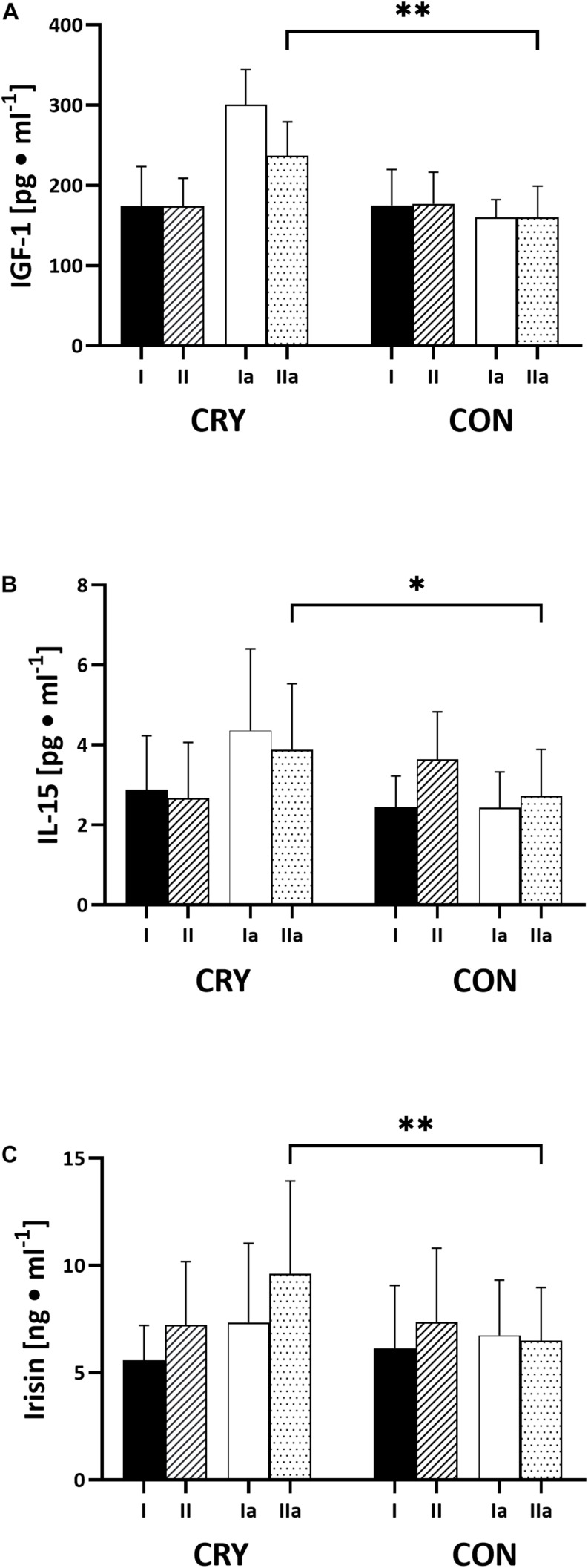
The effect of Special Judo Efficiency Test (SJET) performed before and post 2 weeks of intervention. Comparison serum concentration of resting (I, Ia) and 1 h post SJET values (II, IIa): **(A)** insulin-like growth factor 1 – IGF-1 (*CRY IIa vs CON IIa: p* = 0.01, *effect size* 0.52); **(B)** IL-15, interleukin 15 (*CRY IIa vs CON IIa: p* = 0.03, *effect size* 0.38); **(C)** irisin (*CRY IIa vs CON IIa: p* = 0.00, *effect size* 0.63); CRY – cryostimulation group; CON – control group with passive rest.

The applied intervention did not cause changes in proteins in samples taken 1 h after cryo-session (data not shown). Still, significant differences were observed in serum taken 1 h after the second SJET (Figure 3). These were recorded for IGF-1, IL-15, and irisin concentrations.

### Changes in Amino Acids Concentration

Changes of amino acid serum concentrations are shown in Table 3. Following the specific judo training program and whole body cryostimulation, a significant decrease of proline concentration was observed (Table 3). The magnitude-based inference indicated that the difference between groups was significant and the effect was very likely. Also, a statistically significant decrease of leucine concentration in the CRY group was noted and the effect was likely. The intervention too affected concentrations of isoleucine, valine and tryptophan, but the changes were not significant. There were no significant changes in baseline amino acid serum concentrations before and after the intervention in the CON group.

**TABLE 3:** Amino acid profile at baseline and after 2-week of intervention.

Amino acid [μ*mol*⋅l^–1^]		PRE		POST		SMD	MBI	ANOVA *p* (η${}_{p}^{2}$)
		$\bar{X}$ ± SD	95% CI	$\bar{X}$ ± SD	95% CI	d – Cohena	Practically worthwhile effect	Group × time interaction
**AAs-after deamination form keto acid like alpha-ketoglutarate**								
Proline	CON	149.25 ± 63	83.13 – 215.36	134.35 ± 32.52	100.23 – 168.47	−0,31	Very likely	**0.046 (0.34)**
	CRY	157.98 ± 51.39	104.04 – 211.91	90.99 ± 41.26*	47.69 – 134.29	−1,45		
**AAs-after deamination form keto acid like acetyl-CoA**								
Leucine	CON	85.79 ± 22.92	61.73 – 109.85	104.29 ± 37.37	65.07 – 143.50	0,61	Likely	**0.008 (0.53)**
	CRY	138.51 ± 37.25^#^	99.42 – 177.61	93.43 ± 24.47*	67.75 – 119.10	−1,46		
**Isoleucine**	CON	138.27 ± 62	73.20 – 203.34	121.73 ± 114.67	71.39 – 203.34	−0,19	Unclear	0.718 (0.01)
	CRY	108.10 ± 31.53	68.72 – 147.49	67.65 ± 41.34	24.27 – 111.03	−1,11		
Tryptophan	CON	39.62 ± 13.81	25.13 – 54.12	39.52 ± 7.71	31.43 – 47.61	−0,01	Unclear	0.051 (0.32)
	CRY	50.55 ± 8.93	38.16 – 62.94	32.33 ± 10.03	21.81 – 42.86	−1,82		
**AAs-after deamination form keto acid like fumarate**								
Valine	CON	113.53 ± 47.79	63.43 – 163.73	105.53 ± 46.33	56.91 – 154.16	−0,17	Trivial	0.379 (0.08)
	CRY	133.05 ± 35.75	95.54 – 170.56	95.48 ± 22.31	72.06 – 118.89	−1,29		

PRE, before 2-week of special judo training program; POST, after 2-week of special judo training program; AAs, amino acids; CRY, cryostimulation group; CON, control group; 95%CI, confidence interval; η${}_{p}^{2}$, effect size, partial eta-squared; SMD, standardized mean difference; MBI, magnitude-based inference; #, significant differences between groups; *, significant differences between measurements.

## Discussion

The aim of this study was to determine the effect of the specific judo training, supported by 10 sessions of whole body cryostimulation, on muscle adaptation as reflected in growth factors concentrations, amino acids levels and motor abilities of professional judokas. The applied cryo-intervention caused a significant increase in circulating levels of the two growth factors (BDNF and IGF-1), together with a decrease of the circulating amino acids (proline and leucine) in blood after overnight fasting. Those ameliorations were not observed in judokas in the CON group, who rested passively in-between training units.

Recently published studies, involving martial arts athletes, analyzed only a single application of cold therapy (CWI, 15 min at 10°C) after a single sparring training/combat stimulation unit (Lindsay et al., 2017; Tabben et al., 2018). No studies, however, examined the effect of chronic cold therapy (repeated daily for a certain time) on adaptation to training in professional judo athletes. Malta and co-workers (Malta et al., 2021) based on results obtained from eight papers, reported that effects of CWI (temperature <15°C) are modified by the specificity of strength training (duration, frequency, number of sets). Findings of Roberts et al. (2015) showed that CWI (10 min at 10°C) applied after each unit of high-intensity strength training (12 weeks, 2 units per week, 3 – 5 sets of 8 – 12 RM) substantially attenuated satellite cells’ activity as well as suppressed the acute anabolic signaling in muscle cells after training. This resulted in a small improvement of strength and hypertrophy, mainly in type II muscle fiber. On the other hand, Fyfe and co-workers (Fyfe et al., 2019) observed a negative impact of cold application (15 min at 10°C) performed after resistance training (7 weeks, 3 units per week, 3 – 5 sets of 12 RM) on muscle fibers hypertrophy but had no effect on maximal strength performance. Compared with the above-mentioned studies, in the this study cryostimulation was applied 2 h post rather than immediately after training and the administered temperature was much lower. Based on the fact that CWI (8°C, 4 min) caused a similar decrease of muscle and trunk temperature as a single session of whole body cryostimulation in a special cryo-chamber (Costello et al., 2012), it was possible that the applied cold treatment protocol could decrease judokas’ motor abilities. Meanwhile, the obtained data showed that the applied intervention did not alter muscle performance, sustained muscle function and even slightly improved the SJET results. This is particularly important as our participants represented a very high level of physical capacity.

Alongside high physical capacity demands, mental strength and psychological skills are an integral training component in professional sport. BDNF is one of the growth factors involved in cognitive functions, which has an impact on brain plasticity (Cotman and Berchtold, 2002). Physical activity has been reported to enhance its level (Dinoff et al., 2017), which manifests in boosted brain functions, improved mood and inhibited depression (Teixeira et al., 2010; Phillips, 2017). A recent paper by Schor et al. (2019) reported that combat exercise, like *Randori*, can have a far more significant impact on BDNF release into the bloodstream than other kinds of exercise in elite judo athletes. A single fight induced a much pronounced increase of BDNF in both female and male judokas in comparison to changes after a maximal incremental effort. The authors have explained that judo fights can engage the nervous central system far more than other types of exercise owing to the tactical preparation and quick reactions to sudden changes of action provoked by the opponent (Schor et al., 2019). In our study, a significant difference between groups in the BDNF serum after the intervention was recorded. The 10 sessions of whole body cryostimulation caused an increase in the resting level in BDNF in judokas, yet without changes in body composition or muscle strength. In our previous paper, the specific resistance volleyball training supported by whole body cryostimulation, induced a significant increase of resting BDNF concentration among volleyball players, together with a less pronounced drop of maximal power in the countermovement jump in both female and male athletes (Jaworska et al., 2018). Both studies prove that whole body cryostimulation (9–10 sessions administered daily) combined with resistance training, modified BDNF concentration; however, changes in physical performance depended on the physical workload applied. At the same time, the frequency and duration of breaks in-between cold therapy sessions affect the change of BDNF concentration. For instance, no change in BDNF was noted in untrained students in response to 12 sessions of whole body cryostimulation performed 3 days per week, a day after a strength training session had been completed (Jaworska et al., 2020). IGF-1 is involved in muscle hypertrophy (Adams and McCue, 1998), reduces protein breakdown, and acts as a mediator of anabolic actions in skeletal muscles (Lee et al., 2017). Chronically low concentrations of IGF-1 may reflect an overreaching condition or impaired muscular adaptation to training (Lee et al., 2017). Moreover, IGF-1 is necessary to transform pro-BDNF to BDNF in the central nervous system (Ding et al., 2006). In our study applied procedure induced an increase of IGF-1 only in CRY group, which could have prevented a decline in muscle function.

The obtained data also show a slight increase of IL-15 concentration in blood serum. This myokine, released into the bloodstream in response to exercise (Bazgir et al., 2015), may have a beneficial effect on muscle adaptation by stimulating mitochondrial activity (Quinn et al., 2013). Its elevated level and intensified expression may in turn contribute to an enhanced muscle endurance. Besides the power and strength potential, a high level of aerobic capacity in judokas is particularly relevant for an efficient recovery in-between actions or for an ability to perform technical actions effectively in the last minute of a match (Franchini et al., 2011). Although cryostimulation did not change drastically IL-15 concentration, its level was statistically different after the second Special Judo Efficiency Test.

As evidenced by previous studies, not only exercise (Huh, 2018), but also whole body cryostimulation can be associated with an increase of irisin concentration in the blood (Ziemann et al., 2013). Irisin is considered to be a factor regulating metabolic muscle adaptation, including stimulation of glucose uptake and lipid metabolism (Lee and Jun, 2019). In this study, no statistical alternations in the resting level of irisin were recorded in response to the applied intervention. Similar results were registered by Sliwicka and co-workers (Sliwicka et al., 2020), who noted that whole body cryostimulation had not affected serum irisin concentration in subjects characterized by a high physical fitness level. An interesting correlation between the irisin level and ΔLA after the second SJET in the CRY group was still observed. Currently available data account for a positive impact of irisin on maintaining a balance between glucose uptake in peripheral tissues, including skeletal muscles, and hepatic glucose homeostasis (Arhire et al., 2019). Studies on animal models have shown that irisin stimulated glucose uptake in myocytes and reduced gluconeogenesis in hepatocytes (Mo et al., 2016; Xin et al., 2016). The significant correlation between irisin and ΔLA observed in this study suggest that an increased concentration of circulating irisin may have enhanced lactate removal from blood in response to a high-intensity SJET. During and after exercise, lactate can be utilized via gluconeogenesis, and metabolized to carbon dioxide and water substrate by tissues including brain, skeletal muscle and some others. The actions of irisin on the removal of circulating lactate via gluconeogenesis process is unequivocal (Mo et al., 2016; Xin et al., 2016). A lower concentration of lactate in the CRY group after the high-intensity SJET may indicate that its production decreased, while the utilization increased. Since the cryostimulation intervention was associated with slightly improved scores in the SJET, based mainly on anaerobic glycolysis, lower lactate production can likely be excluded. The role of irisin in lactate removal also cannot be ruled out. A recently published study by Kujach and co-workers (Kujach et al., 2019) demonstrated that a high cumulation of circulating lactate positively correlated with circulating BDNF. It thus cannot be dismissed that the changes in BDNF concentration noted in this study were associated with prefrontal cortex and muscular oxygenation in response to whole body cryostimulation. This observation corresponds with the data obtained by Douzi and co-workers (Douzi et al., 2020), using near-infrared spectroscopy. The authors noted a significant increase in the relative blood perfusion in subjects, who performed the exercise with a cooling vest during submaximal exercise.

Our study also aimed to verify if the applied intervention can affect the amino acid profile. To the best of our knowledge, this is the first study to show an influence of extremely cold temperature on the blood level of proline. The observed change can be explained by two mechanisms.

First, the exposure to cold induces sympathetic-mediated vasoconstriction followed by a reactive vasodilation response that cause the reduction first and the next consequent forced blood flow (Sieroń et al., 2010), resulting in limited availability and subsequently, better uptake of amino acids. Proline is known as the main amino acid component of collagen (Wu et al., 2011) and has an impact on the regeneration process of the articular cartilage matrix (de Paz-Lugo et al., 2018). Poor availability of this crucial amino acid disturbs collagen synthesis, which may increase proneness to osteoarthritis (Wu et al., 2011). In the CRY group, cryostimulation caused a significant drop in the circulating level of this amino acid, whereas in the CON group, the opposite tendency was observed. This may suggest that cryostimulation enhanced the prolinase activity, which was found to recycle proline for collagen resynthesize (Zareba and Palka, 2016). The potential of long-term whole body cryostimulation to enhance collagen structure can make it a useful injury prevention treatment in judo. Permanent grip fight is a crucial part of competitive judo fights; hence, finger injuries are the most common chronic problem among judokas (Strasser et al., 1997). Repetitive and untreated damage to fingers can increase the risk of osteoarthritis, thus, enhanced uptake of proline in response to cold treatment can be considered a recovery method particularly pragmatic for judo players.

Second, the obtained changes can be linked with brown adipose tissue. It is known that exposure to low temperatures stimulates browning fat tissue and its activity (Cannon and Nedergaard, 2004). Recently, Marmol and co-workers (Marmol et al., 2020) revealed that stimulation of activin receptor ALK7 suppressed the expression of Kruppel Like Factor 15 (key regulator of amino acid metabolism) and proline dehydrogenase (amino-acid catabolizing enzyme) in both mouse and human brown adipocytes. This can account for the observed decrease in proline concentration. The applied whole body cryostimulation also caused a drop in the level of branched-chain amino acids in serum after the 2-week intervention. The inter-group difference was most pronounced for leucine, a potential stimulator of muscle protein synthesis and mammalian target of rapamycin complex 1 (mTORC1) signaling human skeletal muscle (Moro et al., 2016). Those findings suggest that cryostimulation may have enhanced the amino acid uptake, supporting the maintenance of motor abilities in judokas after the 2-week training program. Cholewka and co-workers (Cholewka et al., 2012) reported, using thermal imaging, that the extremely cold exposure had a significant effect on skin temperature changes, especially extremities. With a thermal vision camera, the authors demonstrated that one session of whole body cryostimulation (3 min at −120°C) caused a substantial reduction of skin temperature. This rapid body cooling stimulates circulation in the thermoregulation prosses, which affects the constriction of microcirculation vessels and in turn, is quickly followed by vasodilatation due to transmitter substances being released (Sieroń et al., 2010). In this study, whole body cryostimulation caused a significant decrease of proline and leucine levels as well as negative shifts in blood isoleucine and valine. Hence, it can be assumed that by stimulating microcirculation, the applied cold therapy encouraged the peripheral amino acid tissue uptake.

Our study is not without limitations, namely the small sample size of the study group. Our participants were belonged to members of Poland’s national judo team, a selected group accustomed to training with a very high intensity. Extending this group to other, less advanced athletes was hardly possible without affecting the study groups’ core characteristics. In order to credibly interpret the obtained results, we used statistical methods adjusted to the undersized sample size of the study. It is due to these sample size considerations that we could not organize a whole body cryostimulation group in parallel with an untrained group. Future studies, however, could aim to verify the effect of whole body cryostimulation on adaptation training markers in bigger groups of subjects. We would also recommend using in future research not only fitness tests, but also tests to assess the fatigue of the nervous system and the number of mistakes made in tactical tasks, and monitor sleep quality and patterns.

To the best of our knowledge, no reports in the past provided evidence of the effectiveness of whole body cryostimulation as a recovery method in judo. The applied specific training program, combined with 10 sessions of whole body cryostimulation, caused positive, significant changes in BDNF and IGF-1 concentrations as well as the amino acid pool in blood stream post intervention at rest and 1 h after training. The changes in growth factors could be deemed responsible for the muscle function being maintained (not dropping), while the amino acid profile improved. Whole body cryostimulation can thus be considered as a promising physiotherapy procedure for judokas, which can mitigate the overload and overtraining syndrome. Hence, we recommend the use of this treatment during both intense training and competition periods.

## Data Availability Statement

The raw data supporting the conclusions of this article will be made available by the authors, without undue reservation.

## Ethics Statement

The studies involving human participants were reviewed and approved by Bioethics commission in Gdańsk, KB 28/17 on 24 October 2017. The patients/participants provided their written informed consent to participate in this study.

## Author Contributions

JJ, RL, EZ, and PZ designed the study. JJ, RL, EZ, GL, and PZ performed the research. JJ, EZ, KZ, JA, and PZ wrote the manuscript. RL, AJ, KZ, and GL reviewed and editioned the manuscript. All authors contributed to the article and approved the submitted version.

## Conflict of Interest

The authors declare that the research was conducted in the absence of any commercial or financial relationships that could be construed as a potential conflict of interest.

## References

[B1] Adams G. R. McCue S. A. (1998). Localized infusion of IGF-I results in skeletal muscle hypertrophy in rats. *J. Appl. Physiol.* 84 1716–1722. 10.1152/jappl.1998.84.5.1716 9572822

[B2] Arhire L. I. Mihalache L. Covasa M. (2019). Irisin: a hope in understanding and managing obesity and metabolic syndrome. *Front. Endocrinol.* 10:524. 10.3389/fendo.2019.00524 PMC6687775 31428053

[B3] Bazgir B. Salesi M. Koushki M. Amirghofran Z. (2015). Effects of eccentric and concentric emphasized resistance exercise on il-15 serum levels and its relation to inflammatory markers in athletes and non-athletes. *Asian J. Sports Med.* 6:e27980. 10.5812/asjsm.27980 PMC4594145 26448857

[B4] Bouzigon R. Mihailovic T. Lafrance G. Fostel C. (2020). Whole-body cryotherapy accelerates isometric muscle recovery in motocross riders following simulated motocross heats. *Transl. Sports Med.* 3 473–479. 10.1002/tsm2.167

[B5] Bouzigon R. Ravier G. Dugue B. Grappe F. (2018). Thermal sensations during a partial-body cryostimulation exposure in elite basketball players. *J. Hum. Kinet.* 62 55–63. 10.1515/hukin-2017-0158 29922377 PMC6006539

[B6] Cannon B. Nedergaard J. (2004). Brown adipose tissue: function and physiological significance. *Physiol. Rev.* 84 277–359. 10.1152/physrev.00015.2003 14715917

[B7] Cholewka A. Stanek A. Sieron A. Drzazga Z. (2012). Thermography study of skin response due to whole-body cryotherapy. *Skin Res. Technol.* 18 180–187. 10.1111/j.1600-0846.2011.00550.x 21507075

[B8] Cohen J. (1988). *Statistical Power Analysis for the Behavioral Sciences.* Hillsdale, NJ: Lawrence Erlbaum Associates.

[B9] Costello J. T. Culligan K. Selfe J. Donnelly A. E. (2012). Muscle, skin and core temperature after -110 degrees c cold air and 8 degrees c water treatment. *PLoS One* 7:e48190. 10.1371/journal.pone.0048190 23139763 PMC3491015

[B10] Cotman C. W. Berchtold N. C. (2002). Exercise: a behavioral intervention to enhance brain health and plasticity. *Trends Neurosci.* 25 295–301. 10.1016/s0166-2236(02)02143-4 12086747

[B11] de Paz-Lugo P. Lupianez J. A. Melendez-Hevia E. (2018). High glycine concentration increases collagen synthesis by articular chondrocytes in vitro: acute glycine deficiency could be an important cause of osteoarthritis. *Amino Acids* 50 1357–1365. 10.1007/s00726-018-2611-x 30006659 PMC6153947

[B12] Ding Q. Vaynman S. Akhavan M. Ying Z. Gomez-Pinilla F. (2006). Insulin-like growth factor i interfaces with brain-derived neurotrophic factor-mediated synaptic plasticity to modulate aspects of exercise-induced cognitive function. *Neuroscience* 140 823–833. 10.1016/j.neuroscience.2006.02.084 16650607

[B13] Dinoff A. Herrmann N. Swardfager W. Lanctot K. L. (2017). The effect of acute exercise on blood concentrations of brain-derived neurotrophic factor in healthy adults: a meta-analysis. *Eur. J. Neurosci.* 46 1635–1646. 10.1111/ejn.13603 28493624

[B14] Douzi W. Dugue B. Theurot D. Vinches L. Halle S. Dupuy O. (2020). Cooling during exercise may induce benefits linked to improved brain perfusion. *Int. J. Sports Med.* 10.1055/a-1213-5960 Online ahead of print. 32920802

[B15] Franchini E. Takito M. Y. (2014). Olympic preparation in Brazilian judo athletes: description and perceived relevance of training practices. *J. Strength Cond. Res.* 28 1606–1612. 10.1519/jsc.0000000000000300 24149759

[B16] Franchini E. Del Vecchio F. B. Matsushigue K. A. Artioli G. G. (2011). Physiological profiles of elite judo athletes. *Sports Med.* 41 147–166. 10.2165/11538580-000000000-00000 21244106

[B17] Franchini E. Sterkowicz S. Meira C. M. Gomes F. R. Tani G. (2008). Technical variation in a sample of high level judo players. *Percept. Mot. Skills* 106 859–869. 10.2466/pms.106.3.859-869 18712208

[B18] Fyfe J. J. Broatch J. R. Trewin A. J. Hanson E. D. Argus C. K. Garnham A. P. (2019). Cold water immersion attenuates anabolic signaling and skeletal muscle fiber hypertrophy, but not strength gain, following whole-body resistance training. *J. Appl. Physiol.* 127 1403–1418. 10.1152/japplphysiol.00127.2019 31513450

[B19] Garatachea N. Hernandez-Garcia R. Villaverde C. Gonzalez-Gallego J. Torres-Luque G. (2012). Effects of 7-weeks competitive training period on physiological and mental condition of top level judoists. *J. Sports Med. Phys. Fitness* 52 1–10. 22327080

[B20] Gmiat A. Jaworska J. Micielska K. Kortas J. Prusik K. Prusik K. (2018). Improvement of cognitive functions in response to a regular Nordic walking training in elderly women - a change dependent on the training experience. *Exp. Gerontol.* 104 105–112. 10.1016/j.exger.2018.02.006 29432893

[B21] Hopkins W. G. Marshall S. W. Batterham A. M. Hanin J. (2009). Progressive statistics for studies in sports medicine and exercise science. *Med. Sci. Sports Exerc.* 41 3–13. 10.1249/mss.0b013e31818cb278 19092709

[B22] Huh J. Y. (2018). The role of exercise-induced myokines in regulating metabolism. *Arch. Pharm. Res.* 41 14–29. 10.1007/s12272-017-0994-y 29177585

[B23] Jaworska J. Micielska K. Kozlowska M. Wnorowski K. Skrobecki J. Radziminski L. (2018). A 2-week specific volleyball training supported by the whole body cryostimulation protocol induced an increase of growth factors and counteracted deterioration of physical performance. *Front. Physiol.* 9:1711. 10.3389/fphys.2018.01711 PMC6282029 30555349

[B24] Jaworska J. Rodziewicz-Flis E. Kortas J. Kozlowska M. Micielska K. Babinska A. (2020). Short-term resistance training supported by whole-body cryostimulation induced a decrease in myostatin concentration and an increase in isokinetic muscle strength. *Int. J. Environ. Res. Public Health* 17:5496. 10.3390/ijerph17155496 32751455 PMC7432449

[B25] Kujach S. Olek R. A. Byun K. Suwabe K. Sitek E. J. Ziemann E. (2019). Acute sprint interval exercise increases both cognitive functions and peripheral neurotrophic factors in humans: the possible involvement of lactate. *Front. Neurosci.* 13:1455. 10.3389/fnins.2019.01455 PMC6989590 32038149

[B26] Laskowski R. (2007). Próba testowa do oceny specjalnych możliwości wysiłkowych zawodniczek judo (in Polish) - a test trial for assessment of the special performance abilities of female judo athletes. *Sport Wyczynowy* 3 16–27.

[B27] Laskowski R. Antosiewicz J. (2003). Increased adaptability of young judo sportsmen after protein supplementation. *J. Sports Med. Phys. Fitness* 43 342–346. 14625516

[B28] Laskowski R. Ziemann E. Olek R. A. Zembron-Lacny A. (2011). The effect of three days of judo training sessions on the inflammatory response and oxidative stress markers. *J. Hum. Kinet.* 30 65–73. 23487396 10.2478/v10078-011-0074-1 PMC3588647

[B29] Lee E. C. Fragala M. S. Kavouras S. A. Queen R. M. Pryor J. L. Casa D. J. (2017). Biomarkers in sports and exercise: tracking health, performance, and recovery in athletes. *J. Strength Cond. Res.* 31 2920–2937. 10.1519/jsc.0000000000002122 28737585 PMC5640004

[B30] Lee J. H. Jun H. S. (2019). Role of myokines in regulating skeletal muscle mass and function. *Front. Physiol.* 10:42. 10.3389/fphys.2019.00042 PMC6363662 30761018

[B31] Lindsay A. Carr S. Cross S. Petersen C. Lewis J. G. Gieseg S. P. (2017). The physiological response to cold-water immersion following a mixed martial arts training session. *Appl. Physiol. Nutr. Metab* 42 529–536. 10.1139/apnm-2016-0582 28177718

[B32] Lombardi G. Barbaro M. Locatelli M. Banfi G. (2017a). Novel bone metabolism-associated hormones: the importance of the pre-analytical phase for understanding their physiological roles. *Endocrine* 56 460–484. 10.1007/s12020-017-1239-z 28181144

[B33] Lombardi G. Ziemann E. Banfi G. (2017b). Whole-body cryotherapy in athletes: from therapy to stimulation. an updated review of the literature. *Front. Physiol.* 8:258. 10.3389/fphys.2017.00258 PMC5411446 28512432

[B34] Malta E. S. de Lira F. S. Machado F. A. Zago A. S. do Amaral S. L. Zagatto A. M. (2018). Photobiomodulation by led does not alter muscle recovery indicators and presents similar outcomes to cold-water immersion and active recovery. *Front. Physiol.* 9:1948. 10.3389/fphys.2018.01948 PMC6339932 30692939

[B35] Malta E. S. Dutra Y. M. Broatch J. R. Bishop D. J. Zagatto A. M. (2021). The effects of regular cold-water immersion use on training-induced changes in strength and endurance performance: a systematic review with meta-analysis. *Sports Med.* 51 161–174. 10.1007/s40279-020-01362-0 33146851

[B36] Marmol P. Krapacher F. Ibanez C. F. (2020). Control of brown adipose tissue adaptation to nutrient stress by the activin receptor ALK7. *Elife* 9:e54721. 10.7554/eLife.54721 PMC7200161 32366358

[B37] McLester C. N. Nickerson B. S. Kliszczewicz B. M. McLester J. R. (2020). Reliability and agreement of various inbody body composition analyzers as compared to dual-energy x-ray absorptiometry in healthy men and women. *J. Clin. Densitom.* 23 443–450. 10.1016/j.jocd.2018.10.008 30472111

[B38] Meeusen R. Duclos M. Foster C. Fry A. Gleeson M. Nieman D. (2013). Prevention, diagnosis, and treatment of the overtraining syndrome: joint consensus statement of the European college of sport science and the american college of sports medicine. *Med. Sci. Sports Exerc.* 45 186–205. 10.1249/mss.0b013e318279a10a 23247672

[B39] Mo L. Shen J. Liu Q. Zhang Y. Kuang J. Pu S. (2016). Irisin is regulated by car in liver and is a mediator of hepatic glucose and lipid metabolism. *Mol. Endocrinol.* 30 533–542. 10.1210/me.2015-1292 27007446 PMC5414639

[B40] Moro T. Ebert S. M. Adams C. M. Rasmussen B. B. (2016). Amino acid sensing in skeletal muscle. *Trends Endocrinol. Metab* 27 796–806. 10.1016/j.tem.2016.06.010 27444066 PMC5075248

[B41] Murray A. Cardinale M. (2015). Cold applications for recovery in adolescent athletes: a systematic review and meta analysis. *Extrem. Physiol. Med.* 4:17. 10.1186/s13728-015-0035-8 PMC4603811 26464795

[B42] Phillips C. (2017). Brain-derived neurotrophic factor, depression, and physical activity: making the neuroplastic connection. *Neural Plast.* 2017:7260130. 10.1155/2017/7260130 PMC5591905 28928987

[B43] Quinn L. S. Anderson B. G. Conner J. D. Wolden-Hanson T. (2013). IL-15 overexpression promotes endurance, oxidative energy metabolism, and muscle PPARdelta, SIRT1, PGC-1alpha, and PGC-1beta expression in male mice. *Endocrinology* 154 232–245. 10.1210/en.2012-1773 23161867 PMC3529369

[B44] Roberts L. A. Raastad T. Markworth J. F. Figueiredo V. C. Egner I. M. Shield A. (2015). Post-exercise cold water immersion attenuates acute anabolic signalling and long-term adaptations in muscle to strength training. *J. Physiol.* 593 4285–4301. 10.1113/jp270570 26174323 PMC4594298

[B45] Rose C. Edwards K. M. Siegler J. Graham K. Caillaud C. (2017). Whole-body cryotherapy as a recovery technique after exercise: a review of the literature. *Int. J. Sports Med.* 38 1049–1060. 10.1055/s-0043-114861 29161748

[B46] Schaal K. Meur Y. L. E. Louis J. Filliard J. R. Hellard P. (2015). Whole-body cryostimulation limits overreaching in elite synchronized swimmers. *Med. Sci. Sports Exerc.* 47 1416–1425. 10.1249/mss.0000000000000546 25314578

[B47] Schor B. Silva S. G. D. Almeida A. A. Pereira C. A. B. Arida R. M. (2019). Plasma brain-derived neurotrophic factor is higher after combat training (Randori) than incremental ramp test in elite judo athletes. *Braz. J. Med. Biol. Res.* 52:e8154. 10.1590/1414-431X20198154 PMC6459469 30970084

[B48] Sieroń A. Cieślar G. Stanek A. Krzyżanowska-Orlik A. (2010). *Cryotherapy: Theoretical Bases, Biological Effects, Clinical Applications* (Bielsko Biała: α – media press).

[B49] Sikorski W. (2011). New approach to preparation of elite judo athletes to main competition. *J. Combat Sports Martial Arts* 2 57–60. 10.5604/20815735.1047134

[B50] Siqueira A. F. Vieira A. Bottaro M. Ferreira-Junior J. B. Nobrega O. T. de Souza V. C. (2018). Multiple cold-water immersions attenuate muscle damage but not alter systemic inflammation and muscle function recovery: a parallel randomized controlled trial. *Sci. Rep.* 8:10961. 10.1038/s41598-018-28942-5 PMC6053395 30026562

[B51] Sliwicka E. Cison T. Straburzynska-Lupa A. Pilaczynska-Szczesniak L. (2020). Effects of whole-body cryotherapy on 25-hydroxyvitamin D, irisin, myostatin, and interleukin-6 levels in healthy young men of different fitness levels. *Sci. Rep.* 10:6175. 10.1038/s41598-020-63002-x PMC7148349 32277130

[B52] Strasser P. Hauser M. Hauselmann H. J. Michel B. A. Frei A. Stucki G. (1997). [Traumatic finger polyarthrosis in judo athletes: a follow-up study]. *Z Rheumatol.* 56 342–350. 9487650 10.1007/s003930050048

[B53] Tabben M. Ihsan M. Ghoul N. Coquart J. Chaouachi A. Chaabene H. (2018). Cold Water immersion enhanced athletes’ wellness and 10-m short sprint performance 24-h after a simulated mixed martial arts combat. *Front. Physiol.* 9:1542. 10.3389/fphys.2018.01542 PMC6221982 30443221

[B54] Teixeira A. L. Barbosa I. G. Diniz B. S. Kummer A. (2010). Circulating levels of brain-derived neurotrophic factor: correlation with mood, cognition and motor function. *Biomark. Med.* 4 871–887. 10.2217/bmm.10.111 21133708

[B55] Volgyi E. Tylavsky F. A. Lyytikainen A. Suominen H. Alen M. Cheng S. (2008). Assessing body composition with DXA and bioimpedance: effects of obesity, physical activity, and age. *Obesity* 16 700–705. 10.1038/oby.2007.94 18239555

[B56] Wu G. Bazer F. W. Burghardt R. C. Johnson G. A. Kim S. W. Knabe D. A. (2011). Proline and hydroxyproline metabolism: implications for animal and human nutrition. *Amino Acids* 40 1053–1063. 10.1007/s00726-010-0715-z 20697752 PMC3773366

[B57] Xin C. Liu J. Zhang J. Zhu D. Wang H. Xiong L. (2016). Irisin improves fatty acid oxidation and glucose utilization in type 2 diabetes by regulating the AMPK signaling pathway. *Int. J. Obes.* 40 443–451. 10.1038/ijo.2015.199 26403433

[B58] Zareba I. Palka J. (2016). Prolidase-proline dehydrogenase/proline oxidase-collagen biosynthesis axis as a potential interface of apoptosis/autophagy. *Biofactors* 42 341–348. 10.1002/biof.1283 27040799

[B59] Ziemann E. Olek R. A. Grzywacz T. Antosiewicz J. Kujach S. Luszczyk M. (2013). Whole-body cryostimulation as an effective method of reducing low-grade inflammation in obese men. *J. Physiol. Sci.* 63 333–343. 10.1007/s12576-013-0269-4 23744123 PMC3751373

[B60] Ziemann E. Olek R. A. Kujach S. Grzywacz T. Antosiewicz J. Garsztka T. (2012). Five-day whole-body cryostimulation, blood inflammatory markers, and performance in high-ranking professional tennis players. *J. Athl. Train* 47 664–672. 10.4085/1062-6050-47.6.13 23182015 PMC3499891

